# Local Gene Silencing of Monocyte Chemoattractant Protein-1 Prevents Vulnerable Plaque Disruption in Apolipoprotein E-Knockout Mice

**DOI:** 10.1371/journal.pone.0033497

**Published:** 2012-03-12

**Authors:** Xiao Ling Liu, Peng Fei Zhang, Shi Fang Ding, Yan Wang, Mei Zhang, Yu Xia Zhao, Mei Ni, Yun Zhang

**Affiliations:** Key Laboratory of Cardiovascular Remodeling and Function Research, Chinese Ministry of Education and Chinese Ministry of Health, Qilu Hospital, Shandong University, Jinan, Shandong, China; Baker IDI Heart and Diabetes Institute, Australia

## Abstract

Monocyte chemoattractant protein-1 (MCP-1), a CC chemokine (CCL2), has been demonstrated to play important roles in atherosclerosis and becoming an important therapeutic target for atherosclerosis. The present study was undertaken to test the hypothesis that local RNAi of MCP-1 by site-specific delivery of adenovirus-mediated small hairpin RNA (shRNA) may enhance plaque stability and prevent plaque disruption in ApoE−/− mice. We designed an adenovirus-mediated shRNA against mouse MCP-1 (rAd5-MCP-1-shRNA). Male apolipoprotein E-knockout (ApoE−/−) mice (n = 120) were fed a high-fat diet and vulnerable plaques were induced by perivascular placement of constrictive collars around the carotid artery, intraperitoneal injection of lipopolysaccharide and stress stimulation. Mice were randomly divided into RNA interference (Ad-MCP-1i) group receiving local treatment of rAd5-MCP-1-shRNA suspension, Ad-EGFP group receiving treatment of rAd5-mediated negative shRNA and mock group receiving treatment of saline. Two weeks after treatment, plaque disruption rates were significantly lower in the Ad-MCP-1i group than in the Ad-EGFP group (13.3% vs. 60.0%, *P* = 0.01), and local MCP-1 expression was significantly inhibited in the Ad-MCP-1i group confirmed by immunostaining, qRT-PCR and western blot (*P*<0.001). Compared with the Ad-EGFP group, carotid plaques in the Ad-MCP-1i group showed increased levels of collagen and smooth muscle cells, and decreased levels of lipid and macrophages. The expression of inflammatory cytokines and activities of matrix metalloproteinases (MMPs) were lower in the Ad-MCP-1i group than in the Ad-EGFP group. In conclusion, site-specific delivery of adenoviral-mediated shRNA targeting mouse MCP-1 downregulated MCP-1 expression, turned a vulnerable plaque into a more stable plaque phenotype and prevented plaque disruption. A marked suppression of the local inflammatory cytokine expression may be the central mechanism involved.

## Introduction

Atherosclerotic plaque disruption has been identified as the major cause of acute coronary syndrome [1]. A plaque with a large necrotic core and a thin fibrous cap infiltrated by inflammatory cells is vulnerable to disruption. Current evidence demonstrated that inflammation plays a pivotal role in plaque instability and Inflammatory cell recruitment is mediated by various chemotactic cytokines or chemokines [2], [3]. Inhibition of chemokine activity is becoming a therapeutic target in clinical trials of inflammatory disorders [4].

Monocyte chemoattractant protein-1 (MCP-1), a CC chemokine (CCL2), promotes atherosclerosis by recruiting macrophages and monocytes to the vessel wall. MCP-1 was detected in atherosclerotic lesions [5]–[8] and responsible for regulating the local expression of adhesion molecules, interleukin-1 (IL)-1, IL-6 and tissue factor [9], [10]. In recent years, MCP-1 has become an important therapeutic target for atherosclerosis and three treatment approaches have been developed in experimental studies. The first approach involved systemic blocking MCP-1 by injection of an N terminal-deletion mutant of the MCP-1 gene (7ND) and Inoue S et al first showed that 7ND treatment attenuated progression of the atherosclerotic lesions in ApoE−/− mice [11]. Our group first reported that 7ND transfection reversed plaque progression and prevented vulnerable plaques from rupture in a rabbit model [12]. However, it is difficult to translate this approach into clinical application because long-term injection of 7ND may cause hypersensitivity reaction and systemic immunosuppression. The second approach used CCR2 receptor blocker but a recent study found no beneficial effects with this therapy in a mouse model of atherosclerosis, possibly because the CCR2 receptor may have other functions beyond the control of monocyte emigration [13]. The third approach entails local gene silencing of MCP-1 using RNA interference (RNAi) and if this technique proves effective and safe, it would be feasible to translate RNAi into clinical application by combining small interference RNA with interventional device to locally inhibit MCP-1 gene transcription. However, it is still unknown whether local RNA interference of MCP-1 gene can prevent plaque disruption at an advanced stage of atherosclerosis, mostly because the complete gene sequence of MCP-1 in rabbits is unknown and a mouse model of vulnerable plaque mimic to human counterpart is still lacking. Recently we have successfully developed a novel animal model of vulnerable carotid plaque by introducing stress and lipopolysaccharide (LPS) stimulation to ApoE−/− mice, which shared many characteristics of vulnerable plaque in humans [14]. Pathological studies demonstrated that there was a marked increase in the expression of inflammatory factors in the carotid plaque in this animal model, and among these factors, MCP-1 exhibited the most significant increase in the level of protein expression. These observations suggest that inflammation is likely the central mechanism of plaque instability and MCP-1 might offer a pivotal target for preventing plaque disruption.

In the present study, we hypothesized that local RNAi of MCP-1 by site-specific delivery of adenovirus-mediated small hairpin RNA (shRNA) may enhance plaque stability and prevent plaque disruption in our established mouse model of vulnerable plaque, and a series of experiments were designed and performed to test this hypothesis.

## Materials and Methods

### Ethics Statement

The animal experimental protocol complied with the Animal Management Rules of the Chinese Ministry of Health (document No 55, 2001) and was approved by Animal Care Committee of Shandong University.

### Plasmids and Adenovirus Construction

RNAi Target Finder (GenScript Corp., USA) was used to select the RNAi candidate targets for mouse MCP-1 gene following the guidelines established by Elbashir [15]. The DNA sequences coding the shRNA for MCP-1 (gene ID: BC055070, target sequence 184 to 204) were cloned into the shRNA expression plasmid Pgenesil-1 (Wuhan GeneSil Biotechnology, Wuhan, China), a linear double-stranded DNA consisting of the human U6 promoter. Pgenesil-1-shRNA was cloned into an adenoviral shuttle vector and joined with the recombinant adenovirus type 5 (rAd5) vector by homologous recombination to construct rAd5-MCP-1-shRNA. rAd5-mediated negative shRNA, which contains a nonsense sequence (5′-GAC TTC ATA AGG CGC ATG C-3′), served as a control. Both rAd5-MCP-1-shRNA and the negative control vector encoded the sequences for enhanced green fluorescent protein (EGFP).

### Animal Preparation

**Figure 1:**
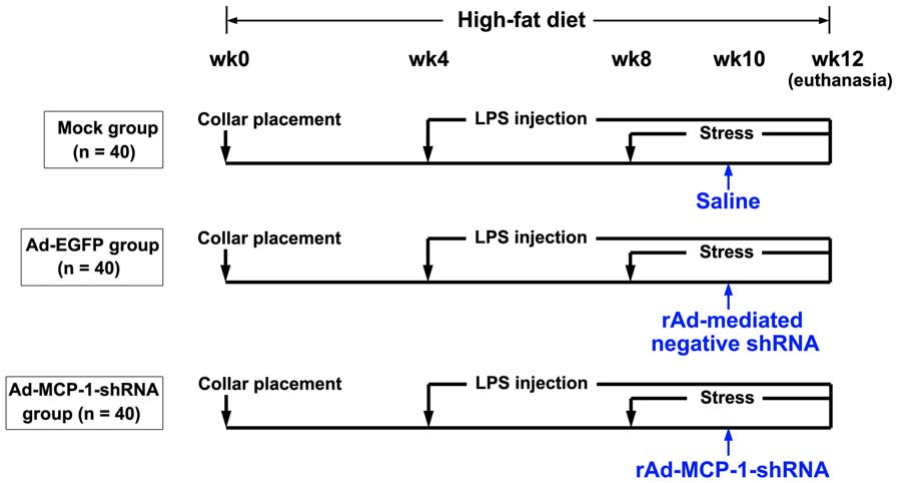
The animal grouping and time line of the experimental protocol. LPS, lipopolysaccharide.

One hundred and twenty ApoE−/− mice (male, 12 weeks old) were obtained from Peking University (Beijing, China). Mice were kept on a 12-hr light/12-h dark cycle, with food and water freely available. All animals were fed a high-fat diet (0.25% cholesterol and 15% cocoa butter) for 12 weeks. The animal grouping and time line of the experimental protocol were shown in Figure 1.

### Animal Model

In our previous study [14], combined stimulation of mental stress and lipopolysaccharide (LPS) was used to create a vulnerable plaque model in ApoE−/− mice fed with high-fat diet. In the present study, we applied this animal model to test our research hypothesis. In brief, carotid atherosclerotic lesions were induced by placement of a restraint perivascular silica collar (3 mm in length and 0.3 mm in internal diameter) around the left common carotid artery [16]. Four weeks after collar placement, mice were injected intraperitoneally with LPS (1 mg/kg in 0.2 ml phosphate-buffered saline, Sigma, USA) twice a week for 8 weeks to induce chronic inflammation. Then, mice were stressed with a 1-h electric foot-shock (0.5 mA for 5 s every 3 min) and noise stimulation (110 dB for 5 s every 3 min) by use of a noise generator (DJ-001, Jianda Technology, China) every day for 4 weeks to induce a hyperhemodynamic status. The intensity of the electric current was adjusted to a minimal level to avoid intolerable pain and skin injury to the mice. To evaluate the effects of stress on hemodynamics, systolic blood pressure (SBP), diastolic blood pressure (DBP) and heart rate (HR) were measured at week 8 before stress stimulation, at week 10 before adenovirus transfection and at week 12 at the end of the experiment, respectively, using a noninvasive tail-cuff system (Softron BP-98A, Tokyo, Japan).

### rAd-MCP1-shRNA Delivery *in vivo*

Ten weeks after collar placement, all mice were anesthetized again with intraperitoneal injection of pentobarbital sodium (40 mg/kg) and the carotid collars were removed. Mice were randomly divided into three groups for treatment: Ad-MCP-1i group (n = 40), Ad-EGFP group (n = 40) and mock group (n = 40). In the Ad-MCP-1i group, 1.25×10^9^ pfu of rAd5-MCP-1-shRNA suspension was gently spread around the adventitia of the left common carotid artery and incubated for 20 min at room temperature [17]. The Ad-EGFP group and the mock group underwent the same treatment but with rAd5-mediated negative-control shRNA (1.25×10^9^ pfu) and normal saline, respectively. After gene administration, the skin incision was closed and mice were allowed to recover. Before and for 3 days after the adenovirus delivery, mice were free from any stimulation to ensure their safety.

### Biochemical Analysis

Two weeks after adenovirus transfection, mice were euthanized and weighed, and blood samples were taken from retro-orbital bleeding. The serum levels of total cholesterol, triglycerides, low-density lipoprotein cholesterol, and high-density lipoprotein cholesterol were measured using commercial kits (Roche Diagnostics, Indianapolis, IN). The plasma levels of fibrinogen were measured as previously described [14].

### Histology and Immunostaining

One mouse from each group was euthanized three days after adenovirus transfection and the remaining mice were euthanized two weeks after transfection. The carotid plaques from 15 mice of each treatment group were used for histological and immunochemical analyses. The left ventricle was perfused with phosphate-buffered saline and then with 4% paraformaldehyde under physiological pressure. The left common carotid artery, heart, liver and kidney were dissected and fixed in 4% paraformaldehyde overnight, and then embedded in OCT compound. Serial cryosections, 6 µm thick, were cut every 50 µm along the carotid artery. Sections were analyzed for EGFP expression by fluorescence microscopy after treatment with 0.5% Chicago Sky Blue (Sigma, USA) to inhibit the background autofluorescence of the tissue.

Sections underwent routine staining with hematoxylin and eosin. Collagen was depicted by Masson's-trichrome and Sirius-red staining. Elastin and lipid depositions were identified by Verhoeff staining and Oil-red O staining, respectively. Perl's staining was applied to detect iron deposits, which indicated plaque hemorrhage. The remaining sections were used for immunohistochemical analysis with the following specific antibodies: anti-monocytes/macrophages (MOMA-2, 1∶25, Serotec; UK), anti-tumor necrosis factor (TNF)-α (1∶50, Abcam, UK), anti-matrix metalloproteinase (MMP)-2 (1∶200, Abcam), anti-α-smooth muscle (SM) actin (1∶1000, Sigma), anti-IL-6 (1∶400, Abcam), anti-MMP-9 (1∶500, Abcam), anti-MCP-1 (1∶50, Abcam) and anti-MMP-12 antibody (1∶500, RayBiotech, USA). After incubation with the appropriate horseradish peroxidase-conjugated secondary antibodies (1∶100, Zhongshan Biological Technology, China), sections were incubated with 3′,3′-diaminobenzidine and counterstained with hematoxylin. To locate the site in the plaque where MCP-1 expression was reduced by the local delivery of rAd5-MCP-1-shRNA, sections were immunostained with specific anti-MCP-1 antibody (1∶50, Abcam) for 12 hrs at 4°C and then incubated with Alex 549-conjugated secondary antibody (1∶1000, Invitrogen). Cell nuclei were counterstained by DAPI (Sigma). The expression of MCP-1 and GFP in plaques was observed using a confocal microscope (Zeiss, German). Sections reacted with nonimmune IgG, secondary antibody only, and no primary and secondary antibodies were used as negative controls.

### Morphologic Analysis

In the present study, plaque disruption was defined as discontinuity of the fibrous cap, buried fibrous cap, or plaque hemorrhage [18], [19]. Fibrous cap discontinuity implies a structural defect in the cap, resulting in exposure of the necrotic core to the blood via the gap in the cap. Buried fibrous caps are indicative of healed plaque rupture. Plaque hemorrhage may originate either from the lumen through a ruptured cap or from breakdown of defective microvessels within the plaque. Plaque disruption was examined by analysis of sections at 50-µm intervals. For calculating plaque disruption rate, any ruptured plaque in a given animal was counted only once no matter how many sections from one specimen demonstrated evidence of plaque disruption.

Plaque area was calculated by subtracting the patent lumen area from the area circumscribed by the internal elastic lamina. The severity of atherosclerotic lesions in each vessel was determined by analysis of sections at 50-µm intervals and the site of maximal plaque size was selected for morphologic analysis. The cap thickness and the cap, core and plaque areas were measured by use of an automated image analysis system (Image-Pro Plus 5.0, Media Cybernetics, USA) attached to a color CCD video camera. Positive staining areas were quantified by computer-assisted color-gated measurement, and the ratio of the positive staining area to the plaque area was calculated.

### Real-Time RT-PCR

Total RNA was extracted from the carotid arteries (n = 12 for each group) using Trizol reagent (Invitrogen, USA). Reverse transcription involved use of the M-MLV Kit (Promega, USA) following the manufacturer's protocol. The mRNA expression levels of MCP-1 and alpha 1 subunit of type IV collagen (Col IV-α1) were quantified by real-time RT-PCR with the SYBR Green technology. The sequences of primers (5′ to 3′) were (1) for MCP-1: CAGCCAGATGCAGTTAACGC (forward) and GCCTACTCATTGGGATCATCTTG (reverse); (2) for Col IV-α1: CTGGCACAAAAGGGACGAG (forward) and ACGTGGCCGAGAATTTCACC (reverse). Quantitative values were obtained from the threshold cycle value. The housekeeping gene β-actin was used as an internal control. The target bands were verified by gel electrophoresis. The 2^−ΔΔCT^ method for comparing relative expression between treatments in real-time PCR was applied as described [20].

### Western Blot Analysis

Carotid plaques from 12 mice of each group were used for western blot analysis. Equal amounts of protein (25 ug) obtained from fresh carotid lesions underwent 10%–15% SDS-PAGE, were transferred to nitrocellulose membranes and then probed with the following specific antibodies: anti-MCP-1 antibody (1∶200), anti-collagen IV (1∶800, Abcam), anti-p-p65 (1∶1000, Cell Signaling Technology, USA) and anti-T-p65 (1∶1000, Cell Signaling Technology). The membranes were incubated with peroxidase-labeled secondary antibody (1∶10000) at room temperature for 2 hrs and the blots were developed with enhanced chemiluminescence (Millipore). The intensities of acquired bands were measured by computerized image analysis and normalized to that of β-actin.

### Zymography

MMP-2 and MMP-9 activities were evaluated by gelatin zymography. The vessel proteins were mixed with the same amount of non-reducing sample buffer and resolved by electrophoresis on a sodium dodecylsulfate (SDS) −10% polyacrylamide gels containing gelatin at a concentration of 1 mg/mL. After electrophoresis, the substrate gels were incubated twice with Triton-X-100 solution (2.5%) for 30 minutes each at room temperature to remove SDS. The gels were then incubated in 50 mM Tris-HCl, pH 7.4, 0.15 M NaCl, 5 mM CaCl_2_, 0.02% NaN_3_, and 0.05% Brij 35 for 24 hours at 37°C. The lysis of the substrates in the gels was visualized by staining with 2.5% Coomasie brilliant blue (Sigma). Gelatinolytic activity was detected as white lysis zones against a blue background.

MMP-12 activity was evaluated by casein zymography. Vessel proteins were mixed with the same amount of non-reducing sample buffer and resolved by electrophoresis on a SDS-10% polyacrylamide gel containing α-casein (Sigma) at a concentration of 1 mg/ml. Thereafter, gels were incubated in 2.5% (v/v) Triton X-100 for 30 minutes and soaked for 16 hours in a buffer containing 10 mM CaCl2 and 100 mM NaCl at 37°C. The gels were then stained with Coomassie brillant blue G250. Caseinolytic activity was detected as white lysis zones against a blue background.

### Statistical Analysis

Quantitative values were expressed as mean ± SEM and analyzed by unpaired *t*-test, one-way ANOVA, or repeated measures ANOVA as appropriate. *Post hoc* comparisons were carried out with least significant difference (LSD) test when equal variances were assumed or with Dunnett test when equal variances were not assumed. Qualitative data were analyzed by chi-square test. Correlations were analyzed by linear regression analysis. SPSS 13.0 (SPSS Inc., Chicago, IL, USA) was used for statistical analysis, and a level of *P*<0.05 was considered significant.

## Results

### Hemodynamic and Biochemical Measurements

For each group of mice, SBP, DBP and HR increased significantly at week 10 and 12, i.e., 2 and 4 weeks after stress stimulation, compared with the corresponding parameters measured at week 8 before stress stimulation (all *P*<0.05, Figure S1A–S1C). There were no significant differences in SBP, DBP and HR among the three groups of mice at any of the three time points (Figure S1A–S1C). Similarly, no significant difference was found in body weight, serum levels of total cholesterol, triglycerides, HDL and LDL cholesterol (Table 1), and plasma fibrinogen level (Figure S1D) among the three groups of mice.

**Table 1:** Body weight and serum lipid levels in three groups of mice.

	Mock	Ad-EGFP	Ad-MCP-1i
	(n = 40)	(n = 40)	(n = 40)
Body weight (g)	27.83±1.60	28.97±1.38	28.14±1.74
Total cholesterol (mmol/L)	16.11±4.07	17.47±4.15	16.28±3.91
Triglyceride (mmol/L)	0.54±0.17	0.46±0.13	0.49±0.14
LDL-C (mmol/L)	5.58±1.49	5.22±1.24	5.51±1.16
HDL-C (mmol/L)	1.53±0.27	1.48±0.29	1.53±0.23

Data were mean ± SEM. LDL-C, low-density lipoprotein cholesterol; HDL-C, high-density lipoprotein cholesterol.

### Efficiency of Adenovirus Transfection

**Figure 2:**
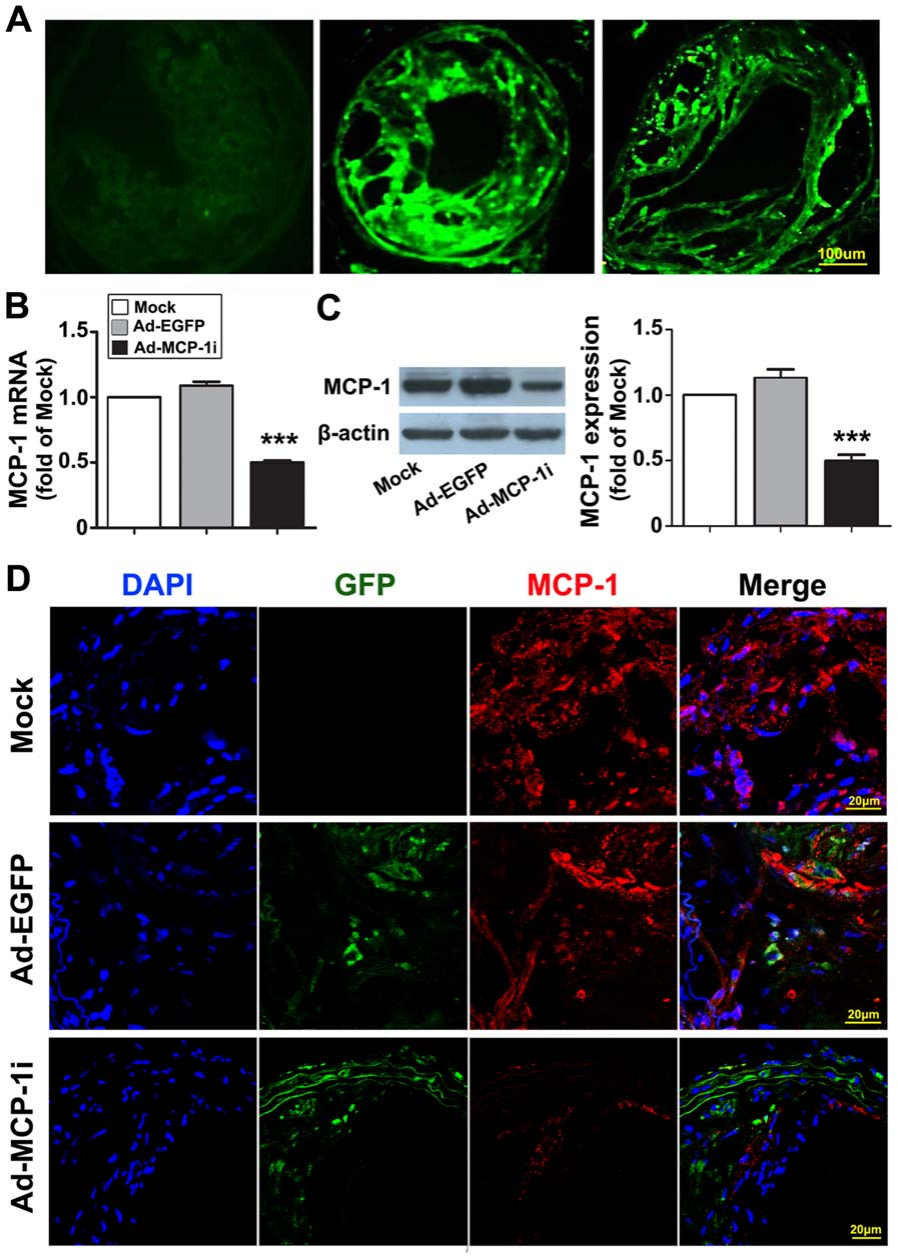
Efficiency of adenovirus transfection in ApoE−/− mice. A, Fluorescence images of the carotid plaques before (left) and three days (middle) and two weeks (right) after adenovirus transfection in the Ad-MCP-1i group. Vessel sections were first treated with SkyBlue 6B to eliminate background autofluorescence of the plaque. Bar = 100 µm; B, mRNA expression of MCP-1 in three groups of mice. ***P<0.001 vs. Ad-EGFP group; C, protein expression of MCP-1 by western blot analysis in three groups of mice. ***P<0.001 vs. Ad-EGFP group; D, Colocalization of the MCP-1 (red) and GFP (green) expression in the carotid plaques in three groups of mice. DAPI (blue) indicated nuclei.

The relative staining area of EGFP in the carotid plaques was 46.0±4.3% and 21.8±2.7% three days and two weeks after shRNA delivery, respectively (Figure 2A). In contrast, the relative staining area of EGFP was only 2.1±0.4% in the liver and undetectable in the heart and kidney 3 days after shRNA delivery. RT-PCR and western blot analyses further demonstrated that the mRNA and protein expression levels of MCP-1 in the carotid plaque were significantly lower in the Ad-MCP-1i group than in the Ad-EGFP group (Figure 2B and 2C). Colocalization of adenovirus vector and MCP-1 expression by immunostaining further confirmed that MCP-1 expression was especially reduced in the GFP-positive lesion in the Ad-MCP-1i group, compared with the Ad-EGFP group (Figure 2D).

### Morphologic Changes of the Carotid Plaque

**Figure 3:**
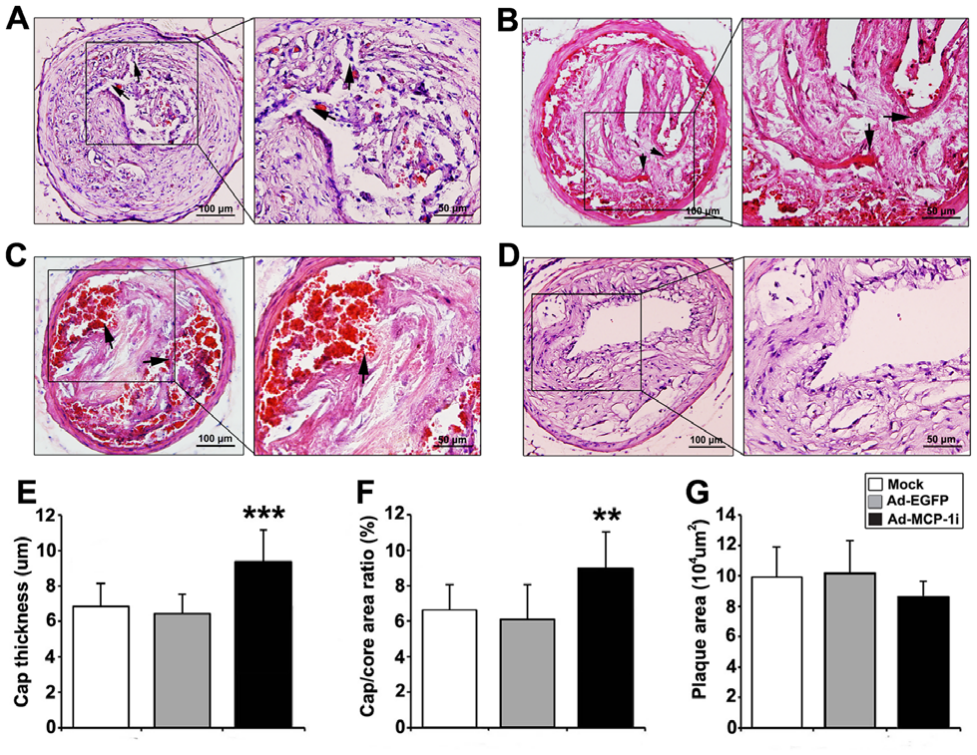
Morphological characteristics of the carotid plaque in three groups of ApoE−/− mice. A, Discontinuity of fibrous cap in the Ad-EGFP group. Arrows indicated the structural defects in the fibrous cap; B, Buried fibrous cap in the Ad-EGFP group. Arrows indicated a layer of fibrous cap within a plaque. C, Plaque hemorrhage in the Ad-EGFP group. Arrows indicated erythrocytes within the plaque; D, An intact carotid plaque in the Ad-MCP-1i group; E–G, quantitative analysis of the fibrous cap thickness (E), the cap to core area ratio (F) and the plaque area (G) in three groups of mice. ** *P*<0.01, *** *P*<0.001, vs. Ad-EGFP group.

Consistent with our previous study, carotid plaques in the mock and Ad-EGFP groups exhibited a high incidence of plaque disruption including fibrous cap discontinuity (Figure 3A), buried fibrous cap (Figure 3B) and plaque hemorrhage (Figure 3C). In contrast, most of the carotid plaques in the Ad-MCP-1i group showed intact fibrous cap (Figure 3D), and the incidence of plaque disruption in the Ad-MCP-1i group (13.3%) was significantly lower than that in the Ad-EGFP group (60.0%, *P*<0.05) or in the mock group (66.7%, *P*<0.05, Table 2). Compared with the Ad-EGFP group, the Ad-MCP-1i group showed significantly higher fibrous cap thickness (9.38±1.74 µm vs. 6.44±1.07 µm; *P*<0.001, Figure 3E) and cap to core area ratio (0.09±0.02 vs. 0.06±0.02; *P* = 0.001, Figure 3F). The plaque area tended to be lower in the Ad-MCP-1i group than the Ad-EGFP and mock groups but the differences did not reach a significant level (*P* = 0.051, Figure 3G).

**Table 2:** Incidence of plaque disruption in three groups of mice.

Plaque complications	Mock	Ad-EGFP	Ad-MCP-1i
	(n = 15)	(n = 15)	(n = 15)
Fibrous cap discontinuity (n)	5	4	1
Buried fibrous cap (n)	4	3	1
Plaque hemorrhage (n)	1	2	0
Total disruption (n, %)	10 (66.7%)	9 (60.0%)	2 (13.3%)*

* *P*<0.05, vs. Ad-EGFP or Mock group.

### Composition Changes of the Carotid Plaque

**Figure 4:**
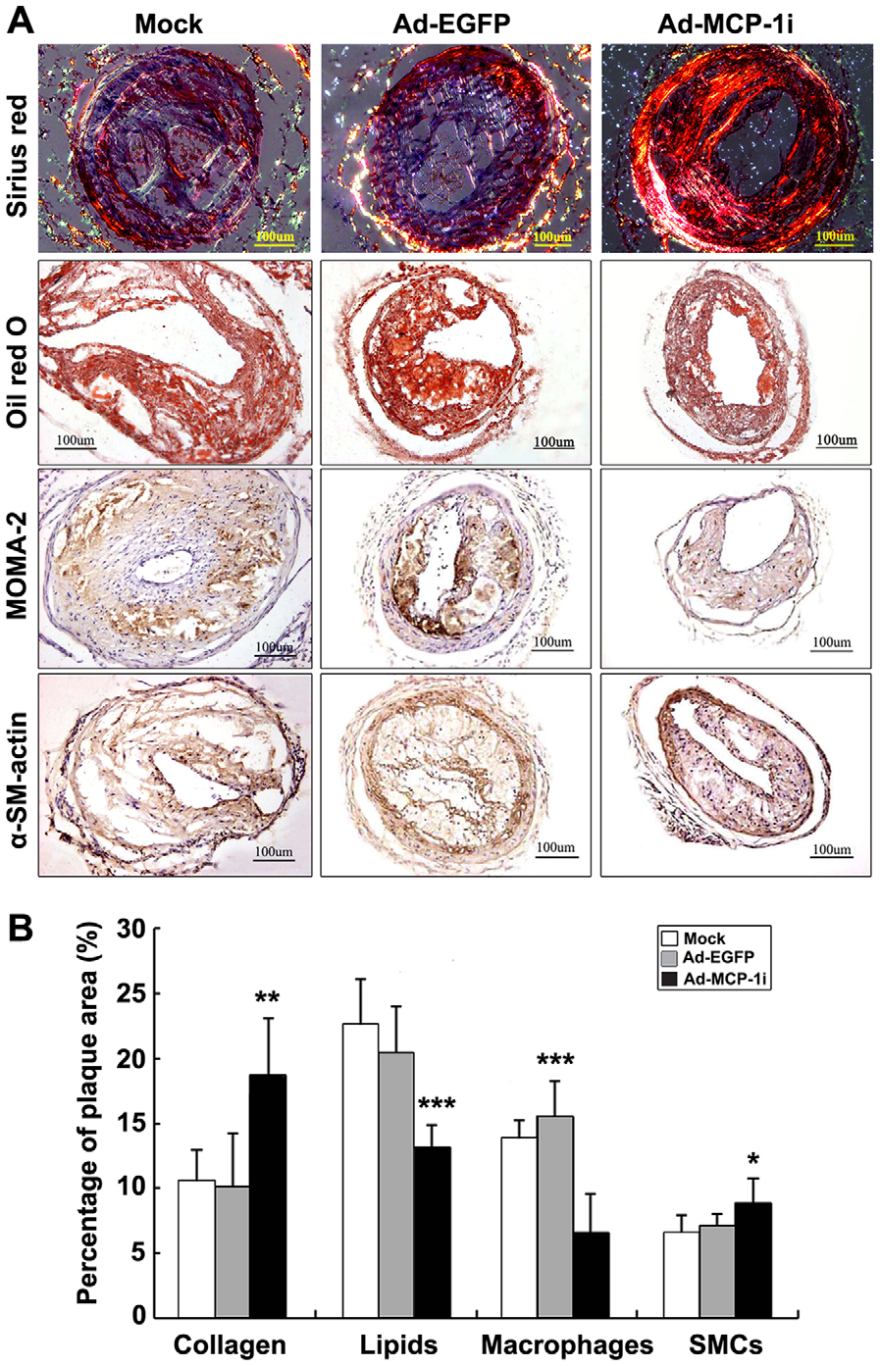
Carotid plaque compositions in three groups of mice. A, Representative Sirius-red staining of collagen, Oil-red O staining of lipids, MOMA-2 immunostaining of macrophages and α-SMC-actin immunostaining in three groups of mice; (B) Quantification of staining results in three groups of mice. * *P*<0.05, ** *P*<0.01, ***P<0.001 vs. Ad-EGFP group.

As shown in Figure 4, carotid plaques in the Ad-MCP-1i group showed a lower content of lipids and macrophages but a higher content of smooth muscle cells and collagen than those in the Ad-EGFP group (*P*<0.05). There was no significant difference in the expression of collagen IV, a major constituent of the basement membrane of the carotid artery, among the three groups (Figure S2A–S2C).

**Figure 5:**
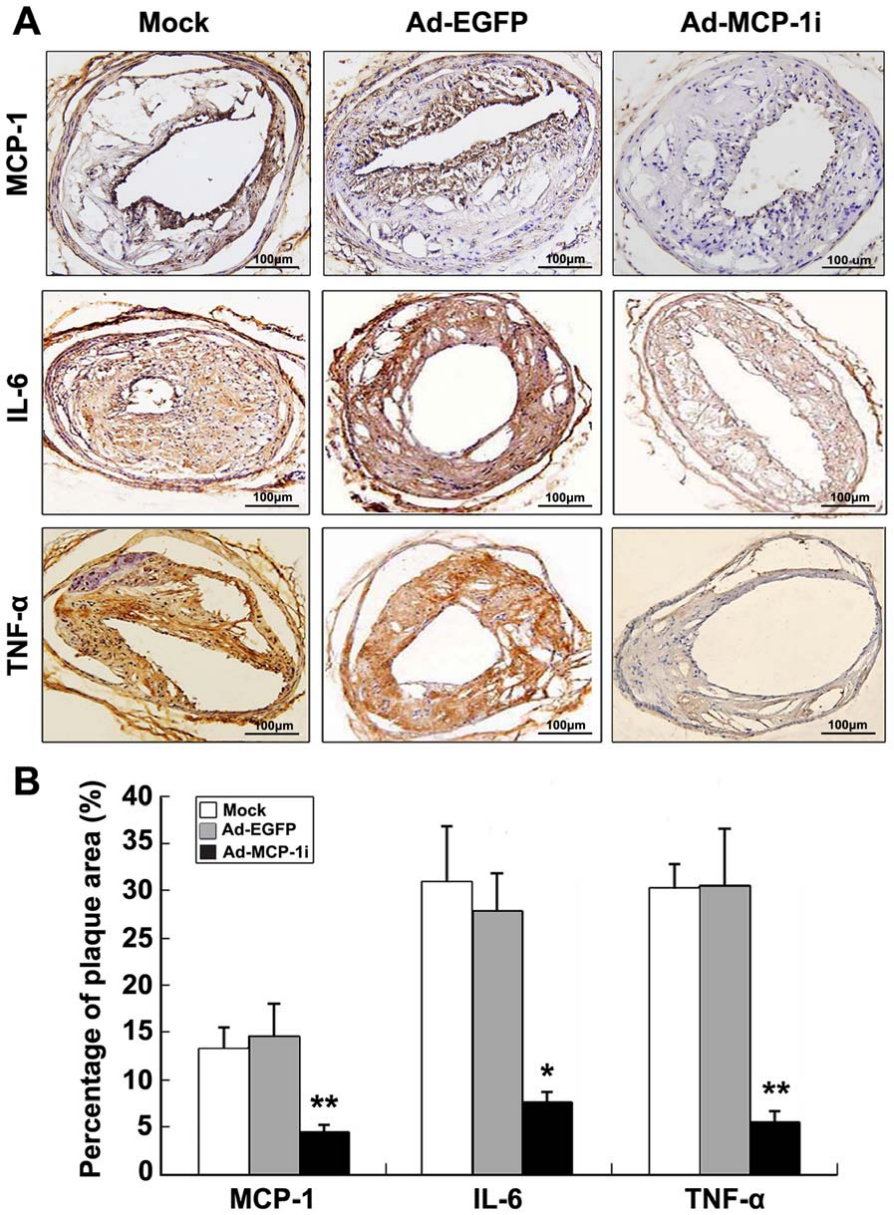
Protein expression of inflammatory cytokines in three groups of mice. A, representative immunostaining of MCP-1, IL-6 and TNF-α in three groups of mice; B, quantitative analysis of MCP-1, IL-6 and TNF-α protein expression in three groups of mice. * *P*<0.01, ** *P*<0.001 vs. Ad-EGFP group.

**Figure 6:**
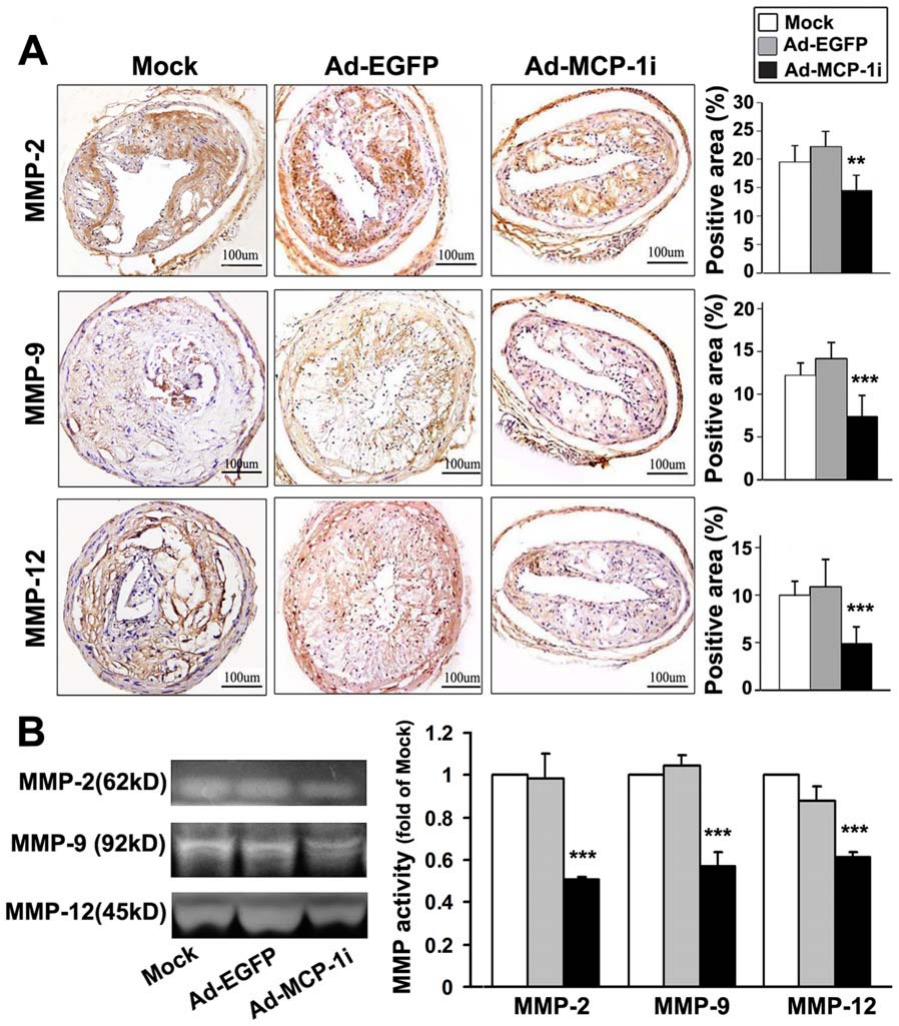
Expression and activation of MMPs in three groups of mice. A, representative immunostaining and quantitative analysis of MMP-2, MMP-9 and MMP-12 expression in three groups of mice. **P<0.01, ***P<0.001 vs. Ad-EGFP group; B, representative zymograms (left panel) and quantitative analysis (right panel) of MMP-2, MMP-9 and MMP-12 activities in three groups of mice. ***P<0.001 vs. Ad-EGFP group.

Plaques from the Ad-MCP-1i group exhibited significantly a lower level of MCP-1 expression than those from the Ad-EGFP group (*P*<0.001; Figure 5). The expression levels of inflammatory cytokines including IL-6 and TNF-α (Figure 5) and matrix metalloproteinases (MMPs) including MMP-2, MMP-9 and MMP-12 (Figure 6A) in the carotid plaque were markedly decreased in the Ad-MCP-1i group as compared with the Ad-EGFP group. Zymography revealed that the activities of MMP-2, MMP-9 and MMP-12 in the carotid plaques were significantly decreased in the Ad-MCP-1i group as compared with the Ad-EGFP group (Figure 6B). In addition, the expression of p-p65 and the ratio of p-p65 to T-p65 were significantly decreased in the Ad-MCP-1i group, as compared with Ad-EGFP group, indicating a suppressed NF-κB activation in the Ad-MCP-1i group (Figure S2B and S2D).

### Correlation between MCP-1 and Other Inflammatory Markers

The protein expression level of MCP-1 was correlated with the relative content of macrophages and the protein expression levels of IL-6, TNF-α, MMP-2, MMP-9 and MMP-12 in the carotid plaque of the three groups of rabbits based on quantitative analysis of immunohistochemical staining. The results showed that the protein expression levels of MCP-1 correlated highly with the relative content of macrophages (r = 0.83, *P*<0.001) and with the protein expression levels of IL-6 (r = 0.93, *P*<0.001), TNF-α (r = 0.92, *P*<0.001), MMP-2 (r = 0.80, *P*<0.001), MMP-9 (r = 0.74, *P*<0.01), and MMP-12 (r = 0.89, *P*<0.001).

## Discussion

The most important finding of the present study was that site-specific delivery of adenoviral-mediated shRNA targeting mouse MCP-1 significantly inhibited MCP-1 expression in the vulnerable carotid plaque of ApoE−/− mice, leading to a more stable plaque phenotype with fewer macrophages, less accumulation of lipids, attenuated expression of inflammatory cytokines and MMPs, more smooth muscle cells and enriched collagen. These plaque composition changes resulted in a more than 4-fold decrease in plaque disruption rate in the RNAi group compared with the control group. Moreover, there were high correlations between MCP-1 expression levels and relative content of macrophages or expression levels of other inflammatory factors in carotid plaques. To the best of our knowledge, this study was the first to report that local gene silencing of MCP-1 may enhance plaque stability and prevent plaque disruption in ApoE−/− mice.

ApoE−/− mice have been widely used as an animal model of atherosclerosis and the murine carotid plaque induced by high-fat diet feeding and paravascular collar placement has been recognized as the most reproducible site for studies of advanced lesions [21]. Recently, we developed a new mouse model of vulnerable carotid plaque by using combinatorial stress stimulation and LPS injection and observed a high incidence of plaque disruption in these mice. The pathophysiological mechanisms underlying plaque disruption in this animal model involved augmented hemodynamic reactivity, blood coagulation and inflammatory processes [14]. In the present study, the plaque disruption rate was 66.7% and 60.0% in the mock group and the Ad-EGFP group, respectively, a finding consistent with our previous report [14].

Among a number of chemokines implicated in atherosclerosis, MCP-1 (i.e., CCL2) is the key chemokine responsible for accumulation of inflammatory cells in atherosclerotic lesions after interaction with its receptor CCR2. This MCP-1–CCR2 interaction leads to activation of G-protein-coupled receptors and then diapedesis of monocytes between endothelial cells [22] Previous studies showed that enhanced expression of MCP-1 gene remarkably promoted whereas its deletion significantly attenuated atherosclerotic lesions [23]–[25]. Aortic atherosclerotic lesions from LDL-receptor/MCP-1 double-knockout mice showed less lipid deposition and fewer macrophages [26], which suggests that MCP-1 is related to plaque instability. In addition, MCP-1 induced tissue-factor expression in smooth muscle cells and T-helper 1 (Th1) cells [10], suggesting that MCP-1 has a procoagulant function. Based on available experimental evidence, it is logical to speculate that MCP-1 offers a promising target for the treatment of vulnerable plaques.

The RNAi method selectively and efficiently silences mRNA for a wide range of proteins [15] and has been used in experimental studies for treating or preventing several diseases [27]–[29]. Two of the most important considerations for developing RNAi as a feasible therapy are first, devising efficient mechanisms for delivery of small-interfering RNA (siRNA) to the target cells *in vivo*, and second, avoiding nonspecific or off-target effects of the siRNA. However, with the systemic delivery method, biodistribution of radiolabeled siRNA in mice demonstrated that most of the siRNA accumulated in the liver and kidneys [30]. Lewis et al showed that systemic coinjection of luciferase reporter plasmids with siRNA luciferase resulted in strong inhibition of luciferase activity not only in the liver but also in kidney, spleen, lung, and pancreas of postnatal mice, which suggests that in mammals, siRNA can be delivered effectively into many organs after systemic application [31]. In order to achieve high-titer rAd5-shRNA in the carotid plaque, we used a site-specific delivery method by spreading the adenovirus suspension around the adventitia of the carotid artery, which was incubated for 20 min at room temperature. Previous studies have demonstrated that site-directed silencing of gene expression is effective and efficient. Furthermore, the inhibitory effects of MCP-1 lasted for two weeks. To assess the nonspecific or off-target effects of this siRNA delivery method, we examined EGFP expression in the heart, liver and kidney and found little expression of EGFP in these organs, which suggested that the siRNA site-specific delivery method was safe and did not affect primary organs other than the targeted vessels.

In our previous study, we found that dominant-negative mutation of MCP-1 (plRES-EGFP-7ND) attenuated atherosclerotic lesion progression and prevented vulnerable aortic plaques from rupture in a rabbit model [12]. In the present study, we obtained similar results in a mouse model of vulnerable plaque and found that Ad-MCP-1-shRNA treatment reduced the MCP-1 gene expression to a greater extension (73.9%) than plRES-EGFP-7ND treatment (60.5%) [12]. Recent studies found that siRNA treatment was as effective as antisense oligonucleotide treatment in both *in vitro* and *in vivo* experiments [32], [33]. Moreover, siRNAs treatment requires no chemical modifications, which results in a high safety profile. Conversely, long-term injection of therapeutic antibodies or recombinant protein infusions may induce systemic immnosuppression or undesirable immune responses against the foreign protein by the host.

In this study, local gene silencing of MCP-1 resulted in 70% reduction in MCP-1 protein expression in the carotid plaque, leading to a marked decrease in the local expression of inflammatory cytokines including IL-6, TNF-α, MMP-2, MMP-9 and MMP-12. These cytokine changes translated into a stable plaque phenotype manifested as decreased macrophages and lipids, and increased smooth muscle cells, collagen and fibrous cap thickness in the carotid plaque. All these changes resulted in a 78% reduction in plaque disruption rate from 60% in the Ad-MCP-1i group to 13% in the Ad-EGFP group, implying that local gene silencing of MCP-1 may effectively prevent disruption of the vulnerable carotid plaque in most ApoE−/− mice. It should be noted that local gene silencing of MCP-1 produced no impact on serum lipid levels and thus the plaque-stabilizing effect of this gene therapy were lipid-independent. These results confirmed our speculation that inflammation is the central mechanism of plaque instability and MCP-1 offers a pivotal target for preventing plaque disruption.

There were several limitations in this study. First, although the plaque-stabilizing effect of MCP-1 gene silencing was encouraging and suppression of inflammatory cytokines was probably the major underlying mechanisms, the detailed signaling pathways were not explored and needs further investigation. Second, different from our previous study in a rabbit model of vulnerable plaque where the plaque burden was significantly reduced by 7ND treatment, local gene silencing of MCP-1 in the present study resulted in an only insignificant reduction in the carotid plaque area. Different animal models and treatment approaches may explain this outcome difference.

In conclusion, in the mouse model of vulnerable plaque, site-specific delivery of adenoviral-mediated shRNA targeting mouse MCP-1 downregulated MCP-1 expression, turned a vulnerable plaque into a more stable plaque phenotype and prevented plaque disruption. The central mechanism may involve a marked decrease in the local expression of inflammatory cytokines. Thus, local gene silencing of MCP-1 may provide an effective approach to the treatment of vulnerable atherosclerotic plaques.

## Supporting Information

Figure S1 **Hemodynamic parameters and plasma fibrinogen levels in three groups of mice.** A–C, systolic blood pressure (A), diastolic blood pressure (B) and heart rate (C) in three groups of mice at week 8 (before stress), week 10 (two weeks after stress), week 12 (four weeks after stress). *P<0.05 vs. week 8 in the mock group; ^#^ *P*<0.05 vs. week 8 in the Ad-EGFP group, ^†^ *P*<0.05, vs. week 8 in the Ad-MCP-1i group; D, plasma fibrinogen levels in three groups of mice.
(TIF)

Figure S2 **Expression of collagen IV and activation of NF kappa B in three groups of mice.** A, mRNA expression of alpha 1 subunit of type IV collagen (Col IV-α1) in three groups of mice; B, Western blot analysis showing protein expression of collagen IV, phospho-NF-κB-p65 (p-p65) and total NF-κB-p65 (T-p65) in three groups of mice. C, quantitative analysis of the collagen IV protein expression in three groups of mice. D, quantitative analysis of the protein expression of p-p65, T-p65 and the ratio of p-p65 to T-p65 (p-p65/T-p65) in three groups of mice. *P<0.05, **P<0.01, vs. Ad-EGFP group.
(TIF)
