# Supplementary figures and images for: Local Gene Silencing of Monocyte Chemoattractant Protein-1 Prevents Vulnerable Plaque Disruption in Apolipoprotein E-Knockout Mice
Source: PLoS One. 2012 Mar 12;7(3):e33497. doi: 10.1371/journal.pone.0033497 (PMC3299803; doi:10.1371/journal.pone.0033497)

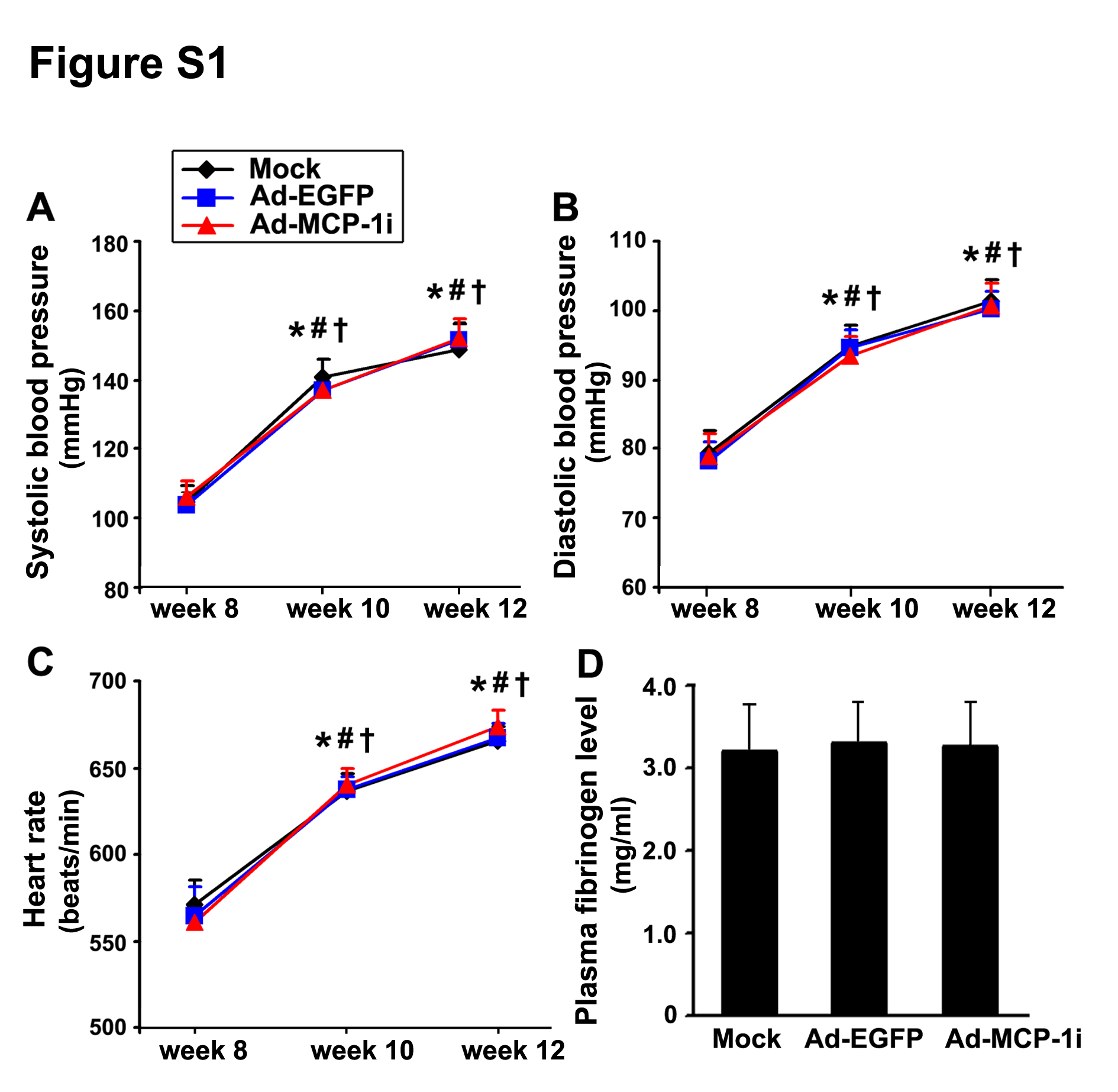

Supplement: Figure S1 — Hemodynamic parameters and plasma fibrinogen levels in three groups of mice. A–C, systolic blood pressure (A), diastolic blood pressure (B) and heart rate (C) in three groups of mice at week 8 (before stress), week 10 (two weeks after stress), week 12 (four weeks after stress). *P<0.05 vs. week 8 in the mock group; # P<0.05 vs. week 8 in the Ad-EGFP group, † P<0.05, vs. week 8 in the Ad-MCP-1i group; D, plasma fibrinogen levels in three groups of mice. (TIF) [file pone.0033497.s001.tif]

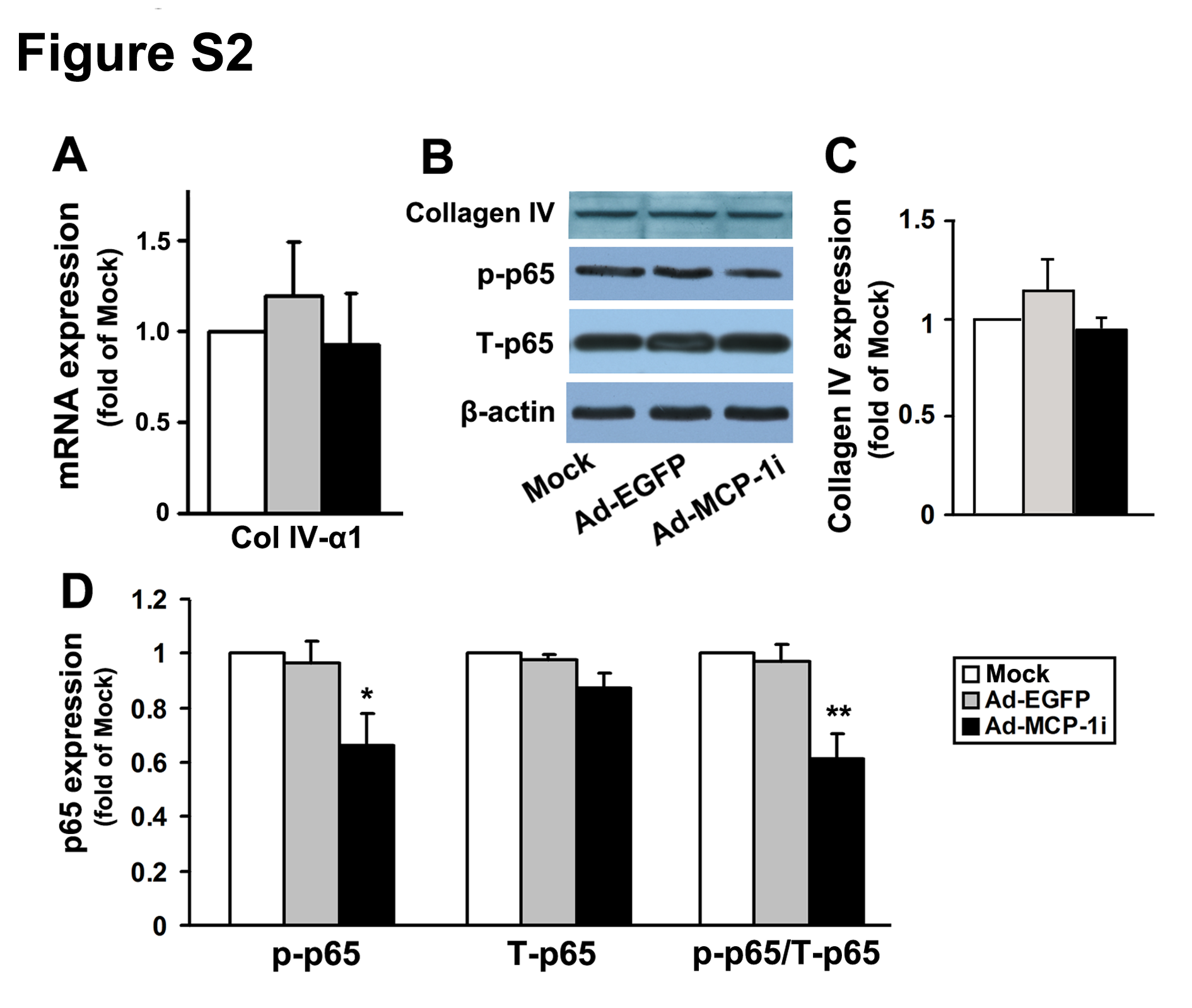

Supplement: Figure S2 — Expression of collagen IV and activation of NF kappa B in three groups of mice. A, mRNA expression of alpha 1 subunit of type IV collagen (Col IV-α1) in three groups of mice; B, Western blot analysis showing protein expression of collagen IV, phospho-NF-κB-p65 (p-p65) and total NF-κB-p65 (T-p65) in three groups of mice. C, quantitative analysis of the collagen IV protein expression in three groups of mice. D, quantitative analysis of the protein expression of p-p65, T-p65 and the ratio of p-p65 to T-p65 (p-p65/T-p65) in three groups of mice. *P<0.05, **P<0.01, vs. Ad-EGFP group. (TIF) [file pone.0033497.s002.tif]
